# Supplementary material for: Effectiveness of Interventions to Manage Difficulties with Breastfeeding for Mothers of Infants under Six Months with Growth Faltering: A Systematic Review Update
Source: Nutrients. 2023 Feb 16;15(4):988. doi: 10.3390/nu15040988 (PMC9961185; doi:10.3390/nu15040988)
Supplement: Supplementary file 1 [file nutrients-15-00988-s001.zip › nutrients-2204749-supplementary.pdf]

Table S1: List of excluded studies

From new search

Exclude on evidence (study design type, trials not yet published) (n= 85)

#	Study	Detail
1	Actrn. Investigating the effect of domperidone on breast milk supply following preterm birth- the SUMMIT study. https://trialsearchwhoint/Trial2aspx?TrialID=ACTRN12621000508875. 2021.-	Trial not yet recruiting
2	Actrn. Giving donor milk instead of formula in moderate-late preterm infants: the GIFT trial. https://trialsearchwhoint/Trial2aspx?TrialID=ACTRN12621000529842. 2021.	Trial not yet recruiting
3	Adhisivam B, Vishnu Bhat B, Banupriya N, Poorna R, Plakkal N, Palanivel C. Impact of human milk banking on neonatal mortality, necrotizing enterocolitis, and exclusive breastfeeding - experience from a tertiary care teaching hospital, south India. The journal of maternal-fetal & neonatal medicine : the official journal of the European Association of Perinatal Medicine, the Federation of Asia and Oceania Perinatal Societies, the International Society of Perinatal Obstetricians. 2019;32(6):902-5.	Pre-post study design. No control group
4	Alshaikh BN, Fenton TR. Additional Insights Into the Use of Preterm Formula Among Neonates. JAMA pediatrics. 2021;175(12):1285-6.	Evidence is a commentary
5	Asztalos EV, Barrington K, Lodha A, Tarnow-Mordi W, Martin A. Lactoferrin infant feeding trial_Canada (LIFT_Canada): protocol for a randomized trial of adding lactoferrin to feeds of very-low-birth-weight preterm infants. BMC pediatrics. 2020;20(1):40.	Protocol
6	Bloomfield FH, Harding JE, Meyer MP, Alsweiler JM, Jiang Y, Wall CR, et al. The DIAMOND trial - DIfferent Approaches to MOderate & late preterm Nutrition: determinants of feed tolerance, body composition and development: protocol of a randomised trial. BMC pediatrics. 2018;18(1):220.	Protocol
7	Bushati C, Chan B, Harmeson O, Alisha, Woodbury A, Yang M, et al. Challenges in Implementing Exclusive Human Milk Diet to Extremely Low-Birth-Weight Infants in a Level III Neonatal Intensive Care Unit. Nutrition in Clinical Practice. 2021;36(6):1198-206.	Study was a comparative cohort study.
8	Calma E, Judkins A, Truong G, Kadri M, Phillips R. Comparison of two commercially available liquid human milk fortifiers for feeding tolerance in premature infants. Pediatrics. 2018;141(1).	Evidence is a conference abstract. No full text available of the study
9	Castellano Y, Cecilia, Castillo B, Beatriz, Muñoz Labián María del C, Ortiz M, et al. Providing very preterm infants with donor human milk led to faster breastfeeding rates but worse biometric gains. Acta Paediatrica. 2019;108(4):766-7.	Pre-post study, with no control group
10	Castro M, Asbury M, Shama S, Stone D, Yoon Eugene W, O'Connor Deborah L, et al. Energy and Fat Intake for Preterm Infants Fed Donor Milk Is Significantly Impacted by Enteral Feeding Method. JPEN Journal of Parenteral & Enteral Nutrition. 2019;43(1):162-5.	Report was on a laboratory observational study

#	Study	Detail
11	Celen R, Tas A, Soylu H. Effect of safe individualized nipple feeding competence (SINC) feeding protocol on weight gain transition to oral feeding and the length of hospitalization in premature infants randomized controlled study. Journal of perinatal medicine. 2019;47:eA302-.	Evidence is a conference abstract. No full text available of the study
12	ChiCtr. Effect of timing of first breast pump stimulation on lactogenesis II and milk production in mothers delivering late preterm infants in a Chinese NICU. https://trialsearchwhoint/Trial2aspx?TrialID=ChiCTR1800017968. 2018.	Trial is in recruiting stage
13	ChiCtr. A randomized controlled trial for comparing sequential and simultaneous bilateral breast pumping for lactation initiation time and maintenance of lactation volume in postpartum non-immediate Breast-feeders. https://trialsearchwhoint/Trial2aspx?TrialID=ChiCTR1800018193. 2018.	Trial is in recruiting stage
14	ChiCtr. A randomized controlled trial for comparing feeding preterm infants fresh versus frozen mother's own milk in the NICU. https://trialsearchwhoint/Trial2aspx?TrialID=ChiCTR1900020577. 2019.	Trial is in recruiting stage
15	Cooke RJ, Griffin I. Iron Balance and Iron Nutritional Status in Preterm Infants During the First Four Months of Life. Journal of pediatric gastroenterology and nutrition. 2021;73(3):403-7.	No control group, observational study
16	Coscia A, Bertino E, Tonetto P, Peila C, Cresi F, Arslanoglu S, et al. Nutritional adequacy of a novel human milk fortifier from donkey milk in feeding preterm infants: study protocol of a randomized controlled clinical trial. Nutrition journal. 2018;17(1).	Protocol
17	Ctri. A clinical trial to study the effects of mobile application based preterm home care program for premature infants. https://trialsearchwhoint/Trial2aspx?TrialID=CTRI/2018/02/012259. 2018.	Trial is in open to recruitment stage.
18	Ctri. A clinical trial to study the effectiveness of a mobile health application for care of Preterms on the improvement of growth parameters and prevention of developmental delays of Preterm infants. https://trialsearchwhoint/Trial2aspx?TrialID=CTRI/2018/06/014368. 2018.	Trial is in open to recruitment stage.
19	Ctri. A Randomized controlled trial to assess the effectiveness of an interventional package in mothers and preterm neonates on preterm neonatal feeding in terms of exclusive breast milk feeding at discharge. https://trialsearchwhoint/Trial2aspx?TrialID=CTRI/2018/02/011950. 2018.	Trial recruitment complete, no published study
20	Ctri. Quantity and fat content of expressed breast milk by Manual expression Vs electric breast pump. https://trialsearchwhoint/Trial2aspx?TrialID=CTRI/2018/05/014229. 2018.	Trial is in open to recruitment stage
21	Ctri. Effect of "Premature Infant Oral Motor Intervention" for early Transition to Oral Feeding in Preterm Infants: a Randomized Controlled Trial. https://trialsearchwhoint/Trial2aspx?TrialID=CTRI/2019/06/019853. 2019.	Trial is in 'not yet recruiting' stage

#	Study	Detail
22	Ctri. Oromotor intervention to improve transition to full katori spoon feeding in preterm newborns. https://trialsearchwhoint/Trial2aspx?TrialID=CTRI/2021/03/032111. 2021.	Trial is in 'not yet recruiting' stage
23	Ctri. A scientific study to compare between NIFTY cup and Katori-spoon feeding on preterm neonates. https://trialsearchwhoint/Trial2aspx?TrialID=CTRI/2021/06/034252. 2021.	Trial is in 'not yet recruiting' stage
24	Ctri. Effectiveness of music and relaxation on amount of breast milk secretion among preterm mothers. https://trialsearchwhoint/Trial2aspx?TrialID=CTRI/2021/04/033146. 2021.	Trial is in 'not yet recruiting' stage
25	Fu Ting T, Kaplan Heather C, Fields T, Folger Alonzo T, Gordon K, Poindexter Brenda B. Protein Enrichment of Donor Breast Milk and Impact on Growth in Very Low Birth Weight Infants. Nutrients. 2021;13(8):2869.	Observational cohort study
26	Gehl B, Brownell E, Power K, Feinn R, Haines K, Lussier M, et al. Comparison of Types of Breast Milk Fortification at Discharge from the Neonatal Intensive Care Unit and Breast Milk Feeding Rates and Growth at 4 Months Corrected Age. Breastfeeding medicine : the official journal of the Academy of Breastfeeding Medicine. 2020;15(10):655-61.	Retrospective observational study
27	Hariharan D, Veluswami G, Kannappan V, Pangaluru S. Effects of early use of donor human milk on growth, development and neonatal morbidity in very low birth weight infants: comparison to mother's own milk and preterm formula. Journal of pediatric gastroenterology and nutrition. 2019;68:1025-.	Conference abstract
28	Huo J, Wu X, Gu C, Yu Z, Zhang J, Chen X, et al. Using a WeChat mini-program-based lactation consultant intervention to increase the consumption of mother’s own milk by preterm infants in the neonatal intensive care unit: a study protocol for a cluster randomized controlled trial. Trials. 2021;22(1).	Trial protocol
29	Irct20080901001162N. The effect of breastfeeding on the weight of newborns. https://trialsearchwhoint/Trial2aspx?TrialID=IRCT20080901001162N25. 2019.	Trial is in recruiting stage
30	Irct20141118019991N. Evaluating the effect of oropharyngeal colostrum in very low birth weight premature infant. https://trialsearchwhoint/Trial2aspx?TrialID=IRCT20141118019991N2. 2019.	Trial is in pending
31	Irct20150424021917N. Effect of cup, syringe and finger feeding on timing of complete oral feeding and weight gain in preterm infants: a randomized controlled clinical trial. https://trialsearchwhoint/Trial2aspx?TrialID=IRCT20150424021917N11. 2020.	Trial is in recruiting stage
32	Irct20151220025619N. The effect of father's education and participation on the success rate of breastfeeding in mothers with preterm infant. https://trialsearchwhoint/Trial2aspx?TrialID=IRCT20151220025619N3. 2018.	Trial is in pending

#	Study	Detail
33	Irct20180427039436N. The effect of Pimpinella anisum herbal tea on milk volume in mothers and weight gain of preterm infant. https://trialsearchwhoint/Trial2aspx?TrialID=IRCT20180427039436N9. 2021.	Trial is pending
34	Irct20180519039715N. The role of synbiotic in the prevention of necrotizing enterocolitis in preterm neonates. https://trialsearchwhoint/Trial2aspx?TrialID=IRCT20180519039715N2. 2019.	Trial recruitment complete, no published study
35	Irct20180520039729N. Effect of Mava Sylvestric, Chamomile Extract Ointment and Breast milk on pain severity and Recovery of Nipple Sore. https://trialsearchwhoint/Trial2aspx?TrialID=IRCT20180520039729N2. 2020.	Trial recruitment complete, no published study
36	Irct20191114045439N. The effect of lullaby on Volume, fat, total protein and albumin of breast milk in mothers of premature infants admitted to neonatal intensive care unit. https://trialsearchwhoint/Trial2aspx?TrialID=IRCT20191114045439N1. 2020.	Trial is in recruiting stage
37	Irct20201108049304N. The effect of Oketani massage on breastfeeding. https://trialsearchwhoint/Trial2aspx?TrialID=IRCT20201108049304N1. 2020.	Trial is in recruiting stage
38	Irct20210502051155N. Effect of PIOMI combined with music therapy on feeding of premature infants. https://trialsearchwhoint/Trial2aspx?TrialID=IRCT20210502051155N1. 2021.	Trial recruitment complete, no published study
39	Isrctn. Feeding late and moderately preterm infants. https://trialsearchwhoint/Trial2aspx?TrialID=ISRCTN15469594. 2018.	Trial is ongoing
40	Isrctn. Does listening to a guided relaxation recording increase the amount of breastmilk that mothers can express when their babies are born very early? https://trialsearchwhoint/Trial2aspx?TrialID=ISRCTN16356650. 2021.	Trial is ongoing
41	Isrctn. Human milk, nutrition, growth and breastfeeding (Hummingbird). https://trialsearchwhoint/Trial2aspx?TrialID=ISRCTN57339063. 2021.	Trial is ongoing
42	Knake LA, King BC, Gollins LA, Hurst NM, Hagan J, Ford SL, et al. Optimizing the Use of Human Milk Cream Supplement in Very Preterm Infants: Growth and Cost Outcomes. Nutrition in clinical practice : official publication of the American Society for Parenteral and Enteral Nutrition. 2020;35(4):689-96.	Retrospective cohort study at a single quaternary NICU
43	Korff SG, Ross J, Morella K, Taylor SN. Investigation of a Common Clinical Approach to Poor Growth in Preterm Infants. American journal of perinatology. 2020;37(14):1462-6.	Pre-post type cohort study
44	Li L, Wang L, Niu C, Liu C, Lv T, Ji F, et al. Early skin contact combined with mother?s breastfeeding to shorten the process of premature infants ? 30 weeks of gestation to achieve full oral feeding: the study protocol of a randomized controlled trial. Trials. 2021;22(1).	Trial Protocol

#	Study	Detail
45	Lin Y-C, Chen Y-J, Huang C-C, Shieh C-C. Concentrated Preterm Formula as a Liquid Human Milk Fortifier at Initiation Stage in Extremely Low Birth Weight Preterm Infants: Short Term and 2-year Follow-up Outcomes. Nutrients. 2020;12(8):2229.	Retrospective and longitudinal study to review the outcomes of the QI project.
46	Mól N, Zasada M, Kwinta P. Does type of feeding affect body composition in very low birth weight infants? - A prospective cohort study. Pediatrics and neonatology. 2019;60(2):135-40.	Observational cohort study
47	Mwangome M, Murunga S, Kahindi J, Gwiyo P, Mwasho G, Talbert A, et al. Individualized breastfeeding support for acutely ill, malnourished infants under 6 months old. Maternal & Child Nutrition. 2020;16(1):N.PAG-N.PAG.	Observational cohort study
48	Nct. Mother and Late Preterm Lactation Study. https://clinicaltrialsgov/show/NCT03791749. 2019.	Trial is in recruiting stage
49	Nct. FamilyLink and Breastfeeding. https://clinicaltrialsgov/show/NCT03957941. 2019.	Trial is ongoing
50	Nct. Effect of Kangaroo Care on Test Weighing. https://clinicaltrialsgov/show/NCT04252547. 2020.	Trial is ongoing
51	Nct. Effects of a Valved Feeding System in Late-preterm Newborns: the Safe Oral Feeding Trial. https://clinicaltrialsgov/show/NCT04400175. 2020.	Trial is ongoing
52	Nct. Analysis of the Effects of Oral Colostrum Administration in Premature Infants on the Breastfeeding. https://clinicaltrialsgov/show/NCT04454502. 2020.	Trial not yet recruiting; trial status unknown
53	Nct. Effect of Family Integrated Care on the Discharge of Parents With Premature Babies in the Neonatal Intensive Care Unit. https://clinicaltrialsgov/show/NCT04478162. 2020.	Trial not yet recruiting; trial status unknown
54	Nct. Reducing Disparity in Receipt of Mother's Own Milk in Very Low Birth Weight Infants. https://clinicaltrialsgov/show/NCT04540575. 2020.	Trial is ongoing
55	Nct. Growth of Preterm Infants Fed a Infant Milk Formula Containing High Versus Low Amounts of Beta-palmitate. https://clinicaltrialsgov/show/NCT04541095. 2020.	Trial is ongoing
56	Nct. ELICIT 2.0: pilot Study of the Effect of Maternal Protein Supplementation During Lactation on Childhood Growth. https://clinicaltrialsgov/show/NCT04565314. 2020.	Trial is ongoing
57	Nct. Nicotinamide Riboside and Milk Production in the NICU. https://clinicaltrialsgov/show/NCT04614714. 2020.	Trial not yet recruiting
58	Nct. Targeted Fortification of Pasteurized Donor Human Milk. https://clinicaltrialsgov/show/NCT04640805. 2020.	Trial not yet recruiting
59	Nct. Mobile Strategy to Reduce the Risk of Recurrent Preterm Birth. https://clinicaltrialsgov/show/NCT04663607. 2020.	Trial is in recruiting stage
60	Nct. The Impact of Positioning on Bottle-feeding in Preterm Infants. A Comparative Study PMMHHRI-2018/V/9-SZB. https://clinicaltrialsgov/show/NCT04773613. 2021.	Comparative crossover study. No control group
61	Nct. the Effect of White Noise on Sucking and Baby Comfort. https://clinicaltrialsgov/show/NCT04807647. 2021.	Trial is ongoing
62	Nct. Human Milk Fortification With Adjustable Versus Targeted Method. https://clinicaltrialsgov/show/NCT04809350. 2021.	Trial in 'not yet recruiting' stage

#	Study	Detail
63	Nct. The Effect of Breast Milk Smell on Nutrition in Preterms. https://clinicaltrialsgov/show/NCT04843293. 2021.	Trial is in recruiting stage
64	Nct. Indonesia After Discharge LBW Infant HMF Supplementation Study. https://clinicaltrialsgov/show/NCT04954807. 2021.	Trial completed recruitment, but no published study available.
65	Nct. The Impact of Back and Breast Massage. https://clinicaltrialsgov/show/NCT04980053. 2021.	Trial is in recruiting stage
66	Nct. Weight Gain in Extremely Premature Infant With Target Versus Adjusted Fortification. https://clinicaltrialsgov/show/NCT04982133. 2021.	Trial is in recruiting stage
67	Nct. A Comparison Study of Feeding Prematures in the Side-lying Position on the Right and Left Side - a Pilot Study. https://clinicaltrialsgov/show/NCT04987983. 2021.	Trial is in recruiting stage
68	Nct. The Effect of Oral Feeding Model With a Chronobiological Approach in Preterm Infants. https://clinicaltrialsgov/show/NCT04992819. 2021.	Trial completed, but no published study available.
69	Nct. The Effect of Routine and Random Pacifier Use Methods in Preterm Infants. https://clinicaltrialsgov/show/NCT05036915. 2021.	Trial is in recruiting stage
70	Oltmann L, Bauer L, Gauthier K, Savage R, May KL. KOALA (Kangaroo Oriented Ad Lib Advancement of Breastfeeding) Blocks: creating a bridge to breastfeeding. JPEN Journal of parenteral and enteral nutrition. 2021;45(SUPPL 1):S211-S3.	Conference abstract
71	Oorloff R, Canning A, Hong T, Van Drimmelen M, Chappell L, Ware R, et al. Comparison of teats to support successful oral feeding in preterm infants: a randomised cross over trial. Journal of paediatrics and child health. 2019;55:42-.	Conference abstract
72	Parker L, Sullivan S, Kruger C, Mueller M. Optimal time to initiate breast milk expression in mothers delivering extremely premature infants. Breastfeeding medicine Conference: 19th international society for research in human milk and lactation conference, ISRHML 2018 Japan. 2018;13(7):A-66.	Conference abstract
73	Parker Leslie A, Cacho N, Engelmann C, Benedict J, Wymer S, Michael W, et al. Consumption of Mother's Own Milk by Infants Born Extremely Preterm Following Implementation of a Donor Human Milk Program: A Retrospective Cohort Study. Journal of Pediatrics. 2019;211:33-8.	Observational cohort study
74	Ramaswamy VV. A randomized controlled trial comparing the effect of fortification of human milk with an infant formula powder versus unfortified human milk on the growth of preterm very low birth weight infants. Journal of maternal-fetal & neonatal medicine. 2020;33(21):3719.	Commentary
75	Reitzel N, Dow K, Fucile S. Enhancing breastfeeding establishment in the NICU: a randomized clinical trial of two non-nutritive sucking approaches. Paediatrics and child health (canada). 2019;24:e54-.	Conference abstract
76	Seliga-Siwecka J, Chmielewska A, Jasinska K. Effect of targeted vs standard fortification of breast milk on growth and development of	Protocol

#	Study	Detail
	preterm infants (≤ 32 weeks): study protocol for a randomized controlled trial. Trials. 2020;21(1).	
77	Shukri NHM, Wells JCK, Fewtrell M. Experimental study on mother-infant signalling during breastfeeding: biological and psychological aspects. Maternal & child nutrition. 2020;16.	Conference abstract
78	Sun H, Cao Y, Han S, Cheng R, Liu L, Liu J, et al. A randomized controlled trial protocol comparing the feeds of fresh versus frozen mother's own milk for preterm infants in the NICU. Trials. 2020;21(1):1-11.	Protocol
79	Tctr. Effect of probiotics on the incidence of necrotizing enterocolitis in preterm. https://trialsearchwhoint/Trial2aspx?TrialID=TCTR20200101001. 2019.	Trial is in recruiting stage
80	Tctr. Introduction of complementary foods at 4 vs 6- month corrected age in preterm infants. https://trialsearchwhoint/Trial2aspx?TrialID=TCTR20200316001. 2020.	Trial recruitment complete, no published study
81	Toro-Campos R, Algarin C, Peirano P, Pena M, Murguia-Peniche T, Wu SS, et al. Effect of feeding mode on infant growth and cognitive function: study protocol of the Chilean infant Nutrition randomized controlled Trial (ChiNuT). BMC pediatrics. 2020;20(1).	Protocol
82	Yu J, Wei Z, Wells J, Fewtrell M. Effects of relaxation therapy on maternal psychological status and infant growth following late pre-term and early term delivery: data from a randomised controlled trial. Journal of pediatric gastroenterology and nutrition. 2021;72(SUPPL 1):1216-.	Protocol
83	Yu J, Wells J, Wei Z, Fewtrell M. Effects of relaxation therapy on maternal psychological state, infant growth and gut microbiome: protocol for a randomised controlled trial investigating mother-infant signalling during lactation following late preterm and early term delivery. International breastfeeding journal. 2019;14:50.	Conference abstract
84	Yu J, Wells J, Wei Z, Fewtrell M. Effects of relaxation therapy on maternal psychological states, infant growth and gut microbiome: protocol for a randomised controlled trial investigating mother-infant signalling during lactation following late preterm and early term delivery. Maternal & child nutrition. 2020;16.	Conference abstract
85	Zhou Q, Zhang L, Lee SK, Chen C, Hu XJ, Liu C, et al. A Quality Improvement Initiative to Increase Mother's Own Milk Use in a Chinese Neonatal Intensive Care Unit. Breastfeeding medicine : the official journal of the Academy of Breastfeeding Medicine. 2020;15(4):261-7.	Pre-post study, with no control group

Exclude on outcome (n=44)

#	Study details	Detail
1	Agakidou E, Karagiozoglou-Lampoudi T, Parlapani E, Fletouris DJ, Sarafidis K, Tzimouli V, et al. Modifications of Own Mothers' Milk Fortification Protocol Affect Early Plasma IGF-I and Ghrelin Levels in Preterm Infants. A Randomized Clinical Trial. Nutrients. 2019;11(12).	Outcomes were growth indicators and plasma ghrelin, IGF-I levels. No breastfeeding outcomes

#	Study details	Detail
2	Aggarwal R, Plakkal N, Bhat V. Does oropharyngeal administration of colostrum reduce morbidity and mortality in very preterm infants? A randomised parallel-group controlled trial. Journal of paediatrics and child health. 2021;57(9):1467-72.	Morbidity and mortality related outcomes. No breastfeeding outcome
3	Aguilar-Rodríguez M, León-Castro JC, Álvarez-Cerezo M, Aledón-Andújar N, Escrig-Fernández R, Rodríguez de Dios-Benlloch JL, et al. The Effectiveness of an Oral Sensorimotor Stimulation Protocol for the Early Achievement of Exclusive Oral Feeding in Premature Infants. A Randomized, Controlled Trial. Physical & occupational therapy in pediatrics. 2020;40(4):371-83.	Outcomes were days until full oral feeding, days until first 30% oral intake and until first 100% oral intake. No breastfeeding outcome
4	Angot F, Van Vooren V, Castex C, Glorieux I, Casper C. Behavioral changes in preterm children during nasogastric tube feeding. Comparative study of manual administration by parents versus mechanical administration via electric syringe pump. Early human development. 2020;149:105151.	Outcomes were behavioural signs
5	Atchley CB, Cloud A, Thompson D, Blunt MH, Satnes KJ, Szyld E, et al. Enhanced Protein Diet for Preterm Infants: a Prospective, Randomized, Double-blind, Controlled Trial. Journal of pediatric gastroenterology and nutrition. 2019;69(2):218-23.	Outcomes were anthropometry and fat accretion. No breastfeeding indicators
6	Bulut O, Coban A, Uzunhan O, Ince Z. Effects of Targeted Versus Adjustable Protein Fortification of Breast Milk on Early Growth in Very Low-Birth-Weight Preterm Infants: a Randomized Clinical Trial. Nutrition in clinical practice. 2020;35(2):335-43.	Outcomes were anthropometry and biochemical indicators. No breastfeeding indicators
7	Chinnappan A, Sharma A, Agarwal R, Thukral A, Deorari A, Sankar MJ. Fortification of Breast Milk with Preterm Formula Powder vs Human Milk Fortifier in Preterm Neonates: a Randomized Noninferiority Trial. JAMA pediatrics. 2021.	Outcomes were related to anthropometry and morbidity. No breastfeeding outcome.
8	Dur Ş, Gözen D. Nonnutritive Sucking Before Oral Feeding of Preterm Infants in Turkey: A Randomized Controlled Study. Journal of Pediatric Nursing. 2021;58:e37-e43.	Primary outcomes are heart rate, oxygen saturation and feeding performance. No breastfeeding outcomes
9	Gerges A, Gelfer P, Kennedy K. Randomized trial of earlier versus later oral feeding in very premature infants. Journal of perinatology. 2018;38(6):687-92.	Outcomes were anthropometric and PMA of completion of oral feeding. No breastfeeding outcomes

#	Study details	Detail
10	Hahn-Holbrook J, Little EE, Abbott M. Mothers are more sensitive to infant cues after breastfeeding compared to bottle-feeding with human milk. Hormones and behavior. 2021;136.	Outcome measured is maternal sensitivity, prolactin level and infant behaviour
11	Irct20100411003679N. Evaluating of Two Nutrition Methods Cup Feeding and Finger feeding on Weight Gain and Nutrition Tolerance in Preterm Infants. https://trialsearchwhoint/Trial2aspx?TrialID=IRCT20100411003679N2. 2021.	Outcome are average weight of premature infants and percentage of feeding tolerance of premature infants. No breastfeeding outcome
12	Irct20150731023423N. The Effect of Telephone Supportive Counseling in The Breastfeeding Self-efficacy of Mothers with Preterm Infant 34-36 Weeks. https://trialsearchwhoint/Trial2aspx?TrialID=IRCT20150731023423N13. 2020.	Outcome is maternal self-efficacy
13	Irct20171030037093N. Effect of breast milk enrichment on early infants. https://trialsearchwhoint/Trial2aspx?TrialID=IRCT20171030037093N3. 2019.	Outcomes are anthropometric, no BF outcomes.
14	Isrctn. Preterm milk Fortification in Neonates. https://trialsearchwhoint/Trial2aspx?TrialID=ISRCTN22484792. 2021.	Primary outcome of interest is Faecal calprotectin
15	Jalali SZ, Shiri MR, Shirazi MG. Effect of probiotics on full intestinal feeding in premature infants: a double blind, clinical trial. Iranian journal of pediatrics. 2020;30(3):1-6.	Outcome is time to full intestinal feeding
16	Jensen GB, Ahlsson F, Domellof M, Elfvin A, Naver L, Abrahamsson T. Nordic study on human milk fortification in extremely preterm infants: a randomised controlled trial - The N-forte trial. BMJ open. 2021;11(11).	The primary outcome is a composite of NEC, stage II–III culture-proven sepsis and mortality during the study period
17	Juhl SM, Ye X, Zhou P, Li Y, Iyore EO, Zhang L, et al. Bovine Colostrum for Preterm Infants in the First Days of Life: a Randomized Controlled Pilot Trial. Journal of pediatric gastroenterology and nutrition. 2018;66(3):471-8.	Outcomes are mainly concentrated on feeding intolerance and time to full enteral feeding
18	Lapidaire W, Lucas A, Clayden JD, Clark C, Fewtrell MS. Human milk feeding and cognitive outcome in preterm infants: the role of infection and NEC reduction. Pediatric research. 2021.	Outcomes are related to IEC and IQ
19	Lee EJ. The Effects of Breast Milk Olfactory Stimulation on Physiological Responses, Oral Feeding Progression and Body Weight in Preterm Infants. Journal of Korean Academy of Nursing. 2019;49(2):126-36.	Outcomes were heart rate, saturation rate, gastric residual

#	Study details	Detail
		volume etc and oral feeding
20	Lessen Knoll Brenda S, Daramas T, Drake V. Randomized Controlled Trial of a Prefeeding Oral Motor Therapy and Its Effect on Feeding Improvement in a Thai NICU. JOGNN: Journal of Obstetric, Gynecologic & Neonatal Nursing. 2019;48(2):176-88.	Premature Infant Oral Motor Stimulation intervention to look into mean volume of oral intake and improvement in interval of oral intake
21	Li X-L, Liu Y, Liu M, Yang C-Y, Yang Q-Z. Early Premature Infant Oral Motor Intervention Improved Oral Feeding and Prognosis by Promoting Neurodevelopment. American Journal of Perinatology. 2020;37(6):626-32.	Feeding progression and feeding performance were outcomes
22	Lingyu F, Meili Z, Lianqiang W, Ruiquan W, Bangbang L, Jianfeng Y, et al. Is preterm donor milk better than preterm formula for very-low-birth-weight infants? Food & Nutrition Research. 2021;65:1-7.	Outcome domains are growth, feeding intolerance and sepsis. No BF indicators
23	Nandakumar A, Pournami F, Prabhakar J, Nair PMC, Jain N. Exclusive Breast Milk vs. Hybrid Milk Feeding for Preterm Babies-A Randomized Controlled Trial Comparing Time to Full Feeds. Journal of tropical pediatrics. 2020;66(1):38-45.	The outcomes were related to feeding intolerance, morbidity and mortality. Also, intervention is supplementation of mother's milk with formula feed supplement and comparison is mother's own milk
24	Nct. Preterm Infants May Better Tolerate Warmer Feeds. https://clinicaltrialsgov/show/NCT03743207. 2018.	Outcome is physiological enteral feeding for premature infants by measuring the number of residuals and the residual volumes.
25	Nct. Feeding and Sucking in Preterm Infants. https://clinicaltrialsgov/show/NCT04751903. 2021.	Time to transition to full oral from gavage feeding is reported
26	Nct. The Effect of The Premature Infant Oral Motorınterventıon on Suckıng Capacıty in Preterm Infants. https://clinicaltrialsgov/show/NCT04835155. 2021.	Outcomes were related to feeding progression, transition from

#	Study details	Detail
		gavage to oral feeding and neurodevelopment related
27	Nunes JA, Bianchini EMG, Cunha MC. Oxygen saturation and heart rate in premature: comparison between cup and finger feeding techniques. Codas. 2019;31(6):e20180221.	No breastfeeding related indicators, only weight gain
28	O'Connor DL, Kiss A, Tomlinson C, Bando N, Bayliss A, Campbell DM, et al. Nutrient enrichment of human milk with human and bovine milk--based fortifiers for infants born weighing <1250 g: a randomized clinical trial. American journal of clinical nutrition. 2018;108(1):108-16.	Outcomes were focused on feeding intolerance. Other outcomes were anthropometric, mortality and morbidity.
29	Olhager E, Danielsson I, Sauklyte U, Tornqvist C. Different feeding regimens were not associated with variation in body composition in preterm infants. Journal of maternal-fetal & neonatal medicine. 2021.	Outcomes were anthropometric, body composition and morbidity outcomes. No Breastfeeding related outcomes
30	Parat S, Raza P, Kamleh M, Super D, Groh-Wargo S. Targeted breast milk fortification for very low birth weight (VLBW) infants: nutritional intake, growth outcome and body composition. Nutrients. 2020;12(4).	Outcomes are body composition, milk fortification, and anthropometry.
31	Parker LA, Weaver M, Murgas T, Rj, Shuster J, Li N, et al. Effect of Gastric Residual Evaluation on Enteral Intake in Extremely Preterm Infants: a Randomized Clinical Trial. JAMA pediatrics. 2019;173(6):534-43.	Outcomes were weekly enteral nutrition and anthropometric outcomes
32	Peila C, Spada E, Bertino E, Deantoni S, Percivati F, Moro GE, et al. The "Fortilat" Randomized Clinical Trial Follow-Up: auxological Outcome at 18 Months of Age. Nutrients. 2020;12(12).	Outcomes were auxological outcomes at 18 months. No breastfeeding outcomes
33	Quan M, Wang D, Gou L, Sun Z, Ma J, Zhang L, et al. Individualized Human Milk Fortification to Improve the Growth of Hospitalized Preterm Infants. Nutrition in clinical practice. 2020;35(4):680-8.	Outcomes were protein intake from PN and EN, and WGV and growth related, but no BF outcomes

#	Study details	Detail
34	Rochow N, Fusch G, Ali A, Bhatia A, So HY, Iskander R, et al. Individualized target fortification of breast milk with protein, carbohydrates, and fat for preterm infants: a double-blind randomized controlled trial. Clinical nutrition (Edinburgh, Scotland). 2021;40(1):54-63.	Outcomes were weight gain velocity (g/kg/d) during the first 21 days of intervention, macronutrient intake, nutritive efficiency, weight, head circumference, length, body composition, major morbidities of prematurity, and clinical chemistry on study days 7, 14, and 21. No breastfeeding outcomes
35	Skaaning D, Kronborg H, Brødsgaard A, Solmer R, Pryds O, Carlsen Emma M. No long-term effect of oral stimulation on the intra-oral vacuum in healthy premature infants. Acta Paediatrica. 2020;109(10):2025-32.	Outcome was intra-oral suction strength of infant, no details on breastfeeding. No detailed results on breastfeeding was found
36	Tarnow-Mordi WO, Abdel-Latif ME, Martin A, Pammi M, Robledo K, Manzoni P, et al. The effect of lactoferrin supplementation on death or major morbidity in very low birthweight infants (LIFT): a multicentre, double-blind, randomised controlled trial. The lancet Child & adolescent health. 2020;4(6):444-54.	Hospital survival of low birthweight infants without morbidity by comparing rates of the primary composite outcome of death or major morbidity between study groups
37	Tewari Vishal V, Kumar A, Singhal A, Prakash A, Pillai N, Varghese J. Proportionate Postnatal Growth in Preterm Neonates on Expressed Breast Milk Feeding With Selected Fortification. Nutrition in Clinical Practice. 2020;35(4):715-23.	Outcomes are anthropometric and morbidity outcomes, no breastfeeding outcomes
38	Thanh LQ, Chen YM, Hartweg M, Thi TAN. Effects of higher protein formula with improved fat blend on growth and feeding tolerance in preterm infants: a double-blind, randomized, controlled clinical trial. Cogent medicine. 2018;5(1):36-7.	Intervention aimed at improving anthropometric, biochemical markers and feeding tolerance.

#	Study details	Detail
39	Uygur O, Yalaz M, Can N, Koroglu OA, Kultursay N. Preterm Infants May Better Tolerate Feeds at Temperatures Closer to Freshly Expressed Breast Milk: a Randomized Controlled Trial. Breastfeeding medicine. 2019;14(3):154-8.	Outcomes were physiological enteral feeding for premature infants by measuring the number of residuals and the residual volumes, feeding temperatures, feeding volumes, daily weight gain, transition time to total enteral feeding, need for anti-reflux medical treatment, and body weight at discharge
40	Wang Y, Hung H, Lin C, Wang C, Lin Y, Chang Y. Effect of a Delayed Start to Oral Feeding on Feeding Performance and Physiological Responses in Preterm Infants: a Randomized Clinical Trial. Journal of nursing research (lippincott williams & wilkins). 2018;26(5):324-31.	Outcomes related to feeding performance were total oral intake, feeding duration, and feeding efficiency. No breastfeeding outcomes
41	Wesolowska A, Pietrzak B, Kociszewska-Najman B, Wielgos M, Czajkowski K, Wietrak E, et al. Barley malt-based composition as a galactagogue - a randomized, controlled trial in preterm mothers. Ginekologia polska. 2021;92(2):118-25.	Use of galactagogue to look into total volume of milk and safety of intervention
42	Yu L, Wang W, Liang Z, Chai H, Lu Y, Liu D. Effects of parenteral nutrition and breastfeeding on the dynamic expression of serum ghrelin in premature infants. International journal of clinical and experimental medicine. 2020;13(3):1643-9.	Outcomes were anthropometric, biochemical and haematological outcomes
43	Yu MX, Zhuang SQ, Gao XY, Tong XM, Yue SJ, Shi LP, et al. Effects of a nutrient-dense formula compared with a post-discharge formula on post-discharge growth of preterm very low birth weight infants with extrauterine growth retardation: a multicentre randomised study in China. Journal of human nutrition and dietetics. 2020;33(4):557-65.	Outcomes are Weight gain, albumin, and globulin levels
44	Zhu L, Gong Y. Analysis on the Application Effect of Abdominal Acupoint Massage on Feeding Intolerance in Premature Infants. Journal of healthcare engineering. 2021;2021:2883597.	Outcomes were incidence of feeding intolerance, MNA nutritional status, and body mass. No breastfeeding outcomes

Exclude on intervention (n=32)

#	Study	Detail
1	Alidad A, Tarameshlu M, Ghelichi L, Haghani H. The effect of non-nutritive sucking combined with oral motor stimulation and oral support on feeding performance in premature infants: a single-blind randomized-clinical trial. Journal of pediatric rehabilitation medicine. 2021;14(3):379-87.	Multiple components in the intervention i.e., NNS+OMS compared with NNS only
2	Armanian AM, Mousavi AS, Salehimehr N. Oral erythromycin and feeding intolerance in preterm infants. Koomesh. 2019;21(1):116-22.	Use of erythromycin, and outcome is feeding intolerance
3	Asztalos EV, Kiss A, daSilva OP, Campbell-Yeo M, Ito S, Knoppert D. Evaluating the Effect of a 14-Day Course of Domperidone on Breast Milk Production: a Per-Protocol Analysis from the EMPOWER Trial. Breastfeeding medicine. 2019;14(2):102-7.	Intervention is use of domperidone
4	Asztalos EV, Kiss A, daSilva OP, Campbell-Yeo M, Ito S, Knoppert D. Role of days postdelivery on breast milk production: a secondary analysis from the EMPOWER trial. International breastfeeding journal. 2019;14:21.	Intervention is use of domperidone
5	Ctri. Mouth cavity application of mothers own milk for prevention of infection in low birth weight babies: a randomised controlled trial. https://trialsearchwhoint/Trial2aspx?TrialID=CTRI/2018/01/011326. 2018.	Intervention is oral care using 0.1 ml of mother's milk to assess late onset sepsis and mortality. Intervention not aimed at improving breastfeeding
6	da Rosa Pereira K, Levy DS, Procianoy RS, Silveira RC. Impact of a pre-feeding oral stimulation program on first feed attempt in preterm infants: double-blind controlled clinical trial. PloS one. 2020;15(9):e0237915.	Intervention included two components- oral stimulation and NNS, which are themselves interventions in other studies. Also, infants were already breastfeeding.
7	Estalella I, San M, Trincado MJ, Maquibar A, Martinez-Indart L, San S. Evaluation of an intervention supporting breastfeeding among late-preterm infants during in-hospital stay. Women and birth. 2018.	Intervention was a multi-component breastfeeding intervention including breastfeeding education, counselling, visit, and phototherapy

#	Study	Detail
8	Govindarajan K, Serane Vikneswari K, Kadirvel K, Palanisamy S. The effects of combined modalities of prefeeding stimulation on feeding progression, length of stay and weight gain in early preterm babies. Journal of Neonatal Nursing. 2020;26(6):330-4.	The intervention was combined modalities of touching and massaging, Oro-motor stimulation, Pacifier stimulation, Swaddling(TOPS stimulation) in addition to NNS and KMC
9	Gun Ja J, Yeon Ran H. Effects of a Breastfeeding Support Program on the Prevalence of Exclusive Breastfeeding and Growth in Late Preterm Infants. Child Health Nursing Research. 2020;26(1):90-7.	Intervention included a combination of web-based breastfeeding education program and practical breastfeeding support.
10	Hei M, Gao X, Li Y, Gao X, Li Z, Xia S, et al. Family Integrated Care for Preterm Infants in China: A Cluster Randomized Controlled Trial. Journal of Pediatrics. 2021;228:36-.	Intervention included multiple components such as site training using bundles of written protocols, printed parent education materials, printed staff training materials etc.
11	Irct20180621040184N. The effect of body massage on the attachment behaviors of premature Infants' mothers. https://trialsearchwhoint/Trial2aspx?TrialID=IRCT20180621040184N1 . 2019.	Intervention is body massage of infant by mothers to assess maternal-infant attachment behaviour
12	Islam MM, Huq S, Hossain MI, Ahmed AMS, Ashworth A, Mollah MAH, et al. Efficacy of F-100, diluted F-100, and infant formula as rehabilitation diet for infants aged < 6 months with severe acute malnutrition: a randomized clinical trial. European journal of nutrition. 2020;59(5):2183-93.	Intervention is use of F-100, diluted F-100 and infant formula to assess weight gain and duration of recovery.
13	Jajoo M, Singh A, Arora N, Bhaskar V, Mandal A. Early Total Versus Gradually Advanced Enteral Nutrition in Stable Very-Low-Birth-Weight Preterm Neonates: a Randomized, Controlled Trial. Indian journal of pediatrics. 2021.	Intervention is early vs gradual enteral feeding and outcome is time to full oral feeds and weight gain. Intervention not aimed at improving breastfeeding

#	Study	Detail
14	Küçük A, İnal S. The Effect of Individualized Developmental Care Practices in Preterm Infants. Complementary medicine research. 2020;27(2):97-104.	Intervention was a combination of breast milk odour, maternal voice, and incubator cloth
15	Kucukoglu S, Yılmaz Kurt F, Aytekin Ozdemir A, Ozcan Z. The Effect of Kangaroo Care on Breastfeeding and Development in Preterm Neonates. Journal of pediatric nursing. 2021;60:e31-e8.	Intervention is KMC
16	Mazumder S, Taneja S, Dube B, Bhatia K, Ghosh R, Shekhar M, et al. Effect of community-initiated kangaroo mother care on survival of infants with low birthweight: a randomised controlled trial. Lancet. 2019;394(10210):1724-36.	Intervention is community KMC
17	McKinney CM, Plange-Rhule G, Ansong D, Cunningham ML, Agyeman I, Coffey PS. A randomized crossover trial comparing the Nifty cup to a medicine cup in preterm infants who have difficulty breastfeeding at Komfo Anokye Teaching Hospital (KATH) in Kumasi, Ghana. PloS one. 2019;14(10):e0223951.	Nifty cup (new feeding cup) vs standard cup assessing caregiver satisfaction and spillage
18	Nct. Effects of Oral Stimulation and Supplemental Nursing System. https://clinicaltrialsgov/show/NCT05087043. 2021.	Combination of oral stimulation and supplemental nursing system used to look into time taken to transition to full breastfeeding and sucking success.
19	O'Brien K, Robson K, Bracht M, Cruz M, Lui K, Alvaro R, et al. Effectiveness of Family Integrated Care in neonatal intensive care units on infant and parent outcomes: a multicentre, multinational, cluster-randomised controlled trial. The lancet Child & adolescent health. 2018;2(4):245-54.	Intervention is family integrated care which includes parent education program, bedside parent coaching etc to assess infant anthropometric and morbidity outcomes
20	Parker LA, Sullivan S, Kruger C, Mueller M. Timing of milk expression following delivery in mothers delivering preterm very low birth weight infants: a randomized trial. Journal of perinatology. 2020;40(8):1236-45.	Interventions were early, immediate and late initiation of milk expression using a hospital-grade breast following delivery to look into amount of mothers' milk consumed
21	Perrella SL, Nancarrow K, Trevenen M, Murray K, Geddes DT, Simmer KN. Effect of vacuum-release teat versus standard teat use on feeding milestones and breastfeeding outcomes in very preterm infants: a randomized controlled trial. PloS one. 2019;14(3):e0214091.	Vacuum released bottle teat compared to standard bottle teat to look into achievement of oral

#	Study	Detail
		feeding and any breastfeeding
22	Pickler Rita H, Meinzen-Derr J, Moore M, Sealschott S, Tepe K. Effect of Tactile Experience During Preterm Infant Feeding on Clinical Outcomes. Nursing Research.69:S21-S8.	Tactile experience during gavage and oral feeding to look into days taken to transition various stages of feeding
23	Rosenthal JL, Sauers-Ford HS, Williams J, Ranu J, Tancredi DJ, Hoffman KR. Virtual Family-Centered Rounds in the Neonatal Intensive Care Unit: a Randomized Controlled Pilot Trial. Academic pediatrics. 2021;21(7):1244-52.	Intervention was virtual family centred rounds in Zoom, looking into feasibility, and potential impact of intervention
24	Shattnawi KK, Al-Ali N. The Effect of Short Duration Skin to Skin Contact on Premature Infants' Physiological and Behavioral Outcomes: A Quasi-Experimental Study. Journal of pediatric nursing. 2019;46:e24-e8.	Intervention was skin to skin contact to assess effect on outcomes such as weight gain, feeding, sleeping, and crying patterns and the occurrence of apneas.
25	Sheng J, Ding Y, Wang J, Zhang J, Qi X, Xia H. The Acceptability, Feasibility, and Effectiveness of Breast Massage Combined with Acupoint Stimulation to Promote the Volume of Human Milk in Mothers with Preterm Infants: a Pilot Study. Evidence-based complementary and alternative medicine. 2021;2021.	Intervention was breast massage and acupoint stimulation looking into feasibility, acceptability
26	Shinde Supriya A, Salunkhe Jyoti A, Mohite V, Salunkhe A, Kakade SV. Effectiveness of Kangaroo Mother Care (KMC) on Lactation among Mothers of Low Birth Weight (LBW) Newborn. International Journal of Nursing Education.11(4):162-4.	Intervention was KMC
27	Tctr. Effect of domperidone on breast milk production in mothers of sick neonates: a randomized, double-blind, placebo-controlled trial. https://trialsearchwhoint/Trial2aspx?TrialID=TCTR20200910008. 2020.	Intervention is use of domperidone.
28	Thabet Amina M, Sayed Zahra A. Effectiveness of the Premature Infant Oral Motor Intervention on Feeding Performance, Duration of Hospital Stay, and Weight of Preterm Neonates in Neonatal Intensive Care Unit: Results From a Randomized Controlled Trial. Dimensions of Critical Care Nursing.40(4):257-65.	Intervention included oral stimulation and NNS with pacifier

#	Study	Detail
29	Travers CP, Wang T, Salas AA, Schofield E, Dills M, Laney D, et al. Higher- or Usual-Volume Feedings in Infants Born Very Preterm: a Randomized Clinical Trial. Journal of pediatrics. 2020;224:66-71.e1.	High volume feed compared to usual volume feed to look into growth velocity, feed tolerance and proportion of exclusive human milk diet
30	Unal S, Demirel N, Bas Ahmet Y, Arifoğlu İ, Erol S, Ulubas I, et al. Impact of Feeding Interval on Time to Achieve Full Oral Feeding in Preterm Infants: A Randomized Trial. Nutrition in Clinical Practice. 2019;34(5):783-8.	2 hour interval compared to 3 hour interval to look into time to achieve full oral feeding
31	Ur R, Iqbal A, Ali W. Role of probiotics in reducing frequency of necrotizing enterocolitis in preterm neonates. Pakistan paediatric journal. 2018;42(3):172-7.	Use of probiotics to assess NEC
32	Varişoğlu Y, Güngör S. The Effects of Listening to Music on Breast Milk Production by Mothers of Premature Newborns in the Neonatal Intensive Care Unit: a Randomized Controlled Study. Breastfeeding medicine. 2020;15(7):465-70.	Music therapy to look into amount of breastmilk increase. Not aimed at improving breastfeeding

Exclude on target population (n=13)

#	Study	Detail
1	Actrn. Effect of boosting breastmilk supply on maternal and infant health. https://trialsearchwhoint/Trial2aspx?TrialID=ACTRN12621001250820. 2021.	Trial excluded preterm babies
2	Ching-Hsueh YEH. The effects of a hospital-based perinatal breastfeeding program on exclusive breastfeeding in Taiwan: a quasi-experimental study. Australian Journal of Advanced Nursing.37(3):20-8.	Includes only healthy term babies
3	Dupont C, Vandenplas Y. Different thickening complexes with pectin in infant anti-regurgitation formula. Acta paediatrica (Oslo, Norway : 1992). 2020;109(3):471-80.	Retrospective analysis of four trials where eligible patients were term or preterm born, presumed healthy, exclusively formula-fed infants, aged <5 months
4	Flaherman VJ, Cabana MD, McCulloch CE, Paul IM. Effect of Early Limited Formula on Breastfeeding Duration in the First Year of Life: a Randomized Clinical Trial. JAMA pediatrics. 2019;173(8):729-35.	Term healthy infants with weight loss who were exclusively breastfed and did not have any difficulties in breastmilk intake.

#	Study	Detail
5	Flaherman VJ, Narayan NR, Hartigan-O'Connor D, Cabana MD, McCulloch CE, Paul IM. The Effect of Early Limited Formula on Breastfeeding, Readmission, and Intestinal Microbiota: a Randomized Clinical Trial. Journal of pediatrics. 2018;196:84-90.e1.	Term healthy infants with weight loss who were exclusively breastfed and did not have any difficulties in breastmilk intake.
6	Irct20180707040370N. The Local Effect of Achillea Millefolium on the Improvement of cracked Nipple. https://trialsearchwhoint/Trial2aspx?TrialID=IRCT20180707040370N1. 2018.	Healthy term born babies
7	Khorana M, Wongsin P, Torbunsupachai R, Kanjanapattanakul W. Effect of Domperidone on Breast Milk Production in Mothers of Sick Neonates: a Randomized, Double-Blinded, Placebo-Controlled Trial. Breastfeeding medicine. 2021;16(3):245-50.	Includes babies who are of normal birth weight also, intervention is a drug - domperidone
8	Lu P, Ye ZQ, Qiu J, Wang XY, Zheng JJ. Acupoint-tuina therapy promotes lactation in postpartum women with insufficient milk production who underwent caesarean sections. Medicine. 2019;98(35):e16456.	Includes only first-time pregnancy carried to the full-term (36–40 weeks); does not mention about babies with growth faltering
9	Mulupuru S, Siddu A, Murki S, Saikiran D, Reddy A. Breast crawl at birth, effect on breastfeeding rate and infant growth in infants delivered at an urban tertiary care public hospital: A randomized controlled trial. Journal of Neonatal Nursing. 2019;25(5):236-9.	Target population were pregnant women. Pre term infants were excluded.
10	Nagarathnamma R, Bhushan P, Dutta T, Ezhil A. A prospective, randomized, placebo-controlled comparative study of amino acid supplementation in lactation insufficiency. Journal of SAFOG. 2020;12(6):408-14.	Mother focussed intervention with no information on infant nutritional status, looking into efficacy of iv amino acid administration. Infant related outcomes were weight and sleep pattern
11	Nordin ZM, Bakar IA, Omar MN, Mahmood A. Effect of consuming lactogenic biscuits formulated with banana (Musa x paradisiaca) flower flour on expressed breast milk (EBM) among lactating working women. Food research. 2020;4(2):294-300.	Infants were born full-term and healthy with the age of 2 to 6 months old and have not yet started weaning diet.
12	Taneja S, Upadhyay RP, Chowdhury R, Kurpad AV, Bhardwaj H, Kumar T, et al. Impact of nutritional interventions among lactating mothers on the growth of their infants in the first 6 months of life: a randomized controlled trial in Delhi, India. American journal of clinical nutrition. 2021;113(4):884-94.	Healthy term born babies and LBW

#	Study	Detail
13	Zhang B, Duan Z, Zhao Y, Williams S, Wall S, Huang L, et al. Intermittent kangaroo mother care and the practice of breastfeeding late preterm infants: results from four hospitals in different provinces of China. International breastfeeding journal. 2020;15(1):64.	Term and preterm babies included

Exclude on comparison (n=1)

#	Study	Details
1	Buldur E, Yalcin B, Terek D, Yalaz M, Altun K, Akisu M, et al. Comparison of the Finger Feeding Method Versus Syringe Feeding Method in Supporting Sucking Skills of Preterm Babies. Breastfeeding medicine. 2020;15(11):703-8.	Finger feeding compared to syringe feeding

From previous review

Exclude on evidence (n=2)

#	Study detail	Detail
1	Colaizy, T.T.; Carlson, S.; Saftlas, A.F.; Morriss, F.H., Jr. Growth in VLBW infants fed predominantly fortified maternal and donor human milk diets: A retrospective cohort study. BMC Pediatr.2012, 12, 124.	Retrospective cohort study
2	Lok, K.Y.W.; Chau, P.H.; Fan, H.S.L.; Chan, K.M.; Chan, B.H.; Fung, G.P.C.; Tarrant, M. Increase in Weight in Low Birth Weight and Very Low Birth Weight Infants Fed Fortified Breast Milk versus Formula Milk: A Retrospective Cohort Study. Nutrients 2017, 9, 520.	Retrospective cohort study examining the effect of feedings with breast milk and formula on the growth and short-term outcomes of preterm infants

Exclude on outcome (n=37)

#	Study details	Details
1	Abrams, S.A.; Schanler, R.J.; Lee, M.L.; Rechtman, D.J. Greater mortality and morbidity in extremely preterm infants fed a diet containing cow milk protein products. Breastfeed. Med. O. J. Acad. Breastfeed. Med. 2014, 9,281–295.	Outcomes were mortality, NEC, growth and duration of parenteral nutrition. No breastfeeding outcomes.
2	Akintorin, S.M.; Kamat, M.; Pildes, R.S.; Kling, P.; Andes, S.; Hill, J.; Pyati, S. A prospective randomized trial of feeding methods in very low birth weight infants. Pediatrics 1997, 100, E4.	Outcomes were feeding intolerance, time to reach full enteral feeds
3	Alan, S.; Atasay, B.; Cakir, U.; Yildiz, D.; Kiliç, A.; Kahvecioglu, D.; Erdeve, O.; Arsan, S. An intention to achieve better postnatal in-hospital-growth for preterm infants: Adjustable protein fortification of human milk. Early Hum. Dev. 2013, 89, 1017–1023.	Outcome was postnatal in-hospital growth of very low birth weight infants
4	Aly, H.; Said, R.N.; Wali, I.E.; Elwakkad, A.; Soliman, Y.S.; Awad, A.R.; Shawky, M.A.; Abu Alam, M.S.; Mohamed, M.A. Medically Graded Honey Supplementation Formula to Preterm Infants as a	Outcomes were anthropometric, CD4 & CD8 counts, bacterial colony count.

	Prebiotic: A Randomized Controlled Trial. J. Pediatr. Gastroenterol. Nutr.2017, 64, 966–970.	
5	Amesz, E.M.; Schaafsma, A.; Cranendonk, A.; Lafeber, H.N. Optimal growth and lower fat mass in preterm infants fed a protein-enriched postdischarge formula. J. Pediatr. Gastroenterol. Nutr.2010, 50, 200–207.	Outcomes were anthropometric and body composition outcomes
6	Arslanoglu, S.; Moro, G.E.; Ziegler, E.E. Adjustable fortification of human milk fed to preterm infants: Does it make a difference? J. Perinatol.O. J. Calif. Perinat. Assoc. 2006, 26, 614–621.	Outcome was anthropometric and biochemical indicators
7	Berseth, C.L.; Harris, C.L.; Wampler, J.L.; Hoffman, D.R.; Diersen-Schade, D.A. Liquid human milk fortifier significantly improves docosahexaenoic and arachidonic acid status in preterm infants. Prostaglandins Leukot. Essent. Fat. Acids 2014, 91, 97–103.	Outcome was fatty acids composition
8	Bhat, B.A.; Gupta, B. Effects of human milk fortification on morbidity factors in very low birth weight infants. Ann. Saudi Med. 2001, 21, 292–305.	Outcomes were related to morbidity status such as sepsis, feeding intolerance and necrotizing enterocolitis
9	Bora, R.; Murthy, N.B. In resource limited areas complete enteral feed in stable very low birth weight infants (1000–1500 g) started within 24 h of life can improve nutritional outcome. J. Matern. Fetal Neonatal Med. 2017,30, 2572–2577.	Outcome measures were feed intolerance(FI) and Necrotising enterocolitis(NEC) in first 21 days of life or discharge from NICU. Mentioned about transfer to complete breastfeeding, but no details in results
10	Cristofalo, E.A.; Schanler, R.J.; Blanco, C.L.; Sullivan, S.; Trawoeger, R.; Kiechl-Kohlendorfer, U.; Dudell, G.; Rechtman, D.J.; Lee, M.L.; Lucas, A.; et al. Randomized trial of exclusive human milk versus preterm formula diets in extremely premature infants. J. Pediatr.2013, 163, 1592–1595.e1.	Outcomes were duration of parenteral nutrition, growth, duration of hospital stay, days of mechanical ventilation and oxygen therapy, and the incidence of LOs, NEC, and retinopathy of prematurity.
11	Erasmus, H.D.; Ludwig-Auser, H.M.; Paterson, P.G.; Sun, D.; Sankaran, K. Enhanced weight gain in preterm infants receiving lactase-treated feeds: A randomized, double-blind, controlled trial. J. Pediatr.2002, 141, 532–537.	Outcomes were anthropometric (weight gain, crown-heel length and occipitofrontal head circumference) and biochemical (serum

		concentrations of protein, albumin, sodium, and potassium) and feeding tolerance
12	Florendo, K.N.; Bellflower, B.; van, Z.; Cooke, R.J. Growth in preterm infants fed either a partially hydrolyzed whey or an intact casein/whey preterm infant formula. J. Perinatol.2009, 29, 106–111.	Outcomes were anthropometry, biochemical markers and nutrient intake
13	Gathwala, G.; Chawla, M.; Gehlaut, V.S.; Gathwala, G.; Chawla, M.; Gehlaut Veena, S. Fortified human milk in the small for gestational age neonate. Indian J. Pediatr.2007, 74, 815–818.	Outcomes were anthropometric and biochemical markers
14	Hair, A.B.; Blanco, C.L.; Moreira, A.; Hawthorne, K.; Lee, M.L.; Rechtman, D.J.; Abrams, S.A. Randomized trial of human milk cream as a supplement to standard fortification of an exclusive human milk-based diet in infants 750-1250 g birth weight. J. Pediatr.2014, 165, 915–920.	Outcomes were growth velocity (weight, length, and HC) and the amount of donor HM derived fortifier used, mortality and morbidity (incidence of sepsis, NEC)
15	Kanmaz, H.G.; Mutlu, B. Human Milk Fortification with Differing Amounts of Fortifier and Its Association with Growth and Metabolic Responses in Preterm Infants. J. Hum. Lact. 2013, 29, 400–405.	Outcomes were anthropometric and biochemical markers
16	Kim, J.H.; Chan, G.; Schanler, R.; Groh-Wargo, S.; Bloom, B.; Dimmit, R.;Williams, L.; Baggs, G.; Barrett-Reis, B. Growth and Tolerance of Preterm Infants Fed a New Extensively Hydrolyzed Liquid Human Milk Fortifier. J. Pediatr. Gastroenterol. Nutr.2015, 61, 665–671.	Outcomes were anthropometric and biochemical markers
17	Kim Eun, J.; Lee Na, M.; Chung, S.-H. A retrospective study on the effects of exclusive donor human milk feeding in a short period after birth on morbidity and growth of preterm infants during hospitalization. Medicine 2017, 96, e7970.	Outcomes were short term morbidities and growth
18	Kumar, N.; Monga, R.; Sampath, V.; Ehrhart, B. Prospective Comparison of Enfamil and Similac Liquid Human Milk Fortifier on Clinical Outcomes in Premature Infants. Am. J. Perinatol.2017, 34, 1411–1416.	Outcomes were clinical outcomes and enteral feeding
19	Lau, C.; Smith, E.O. Interventions to improve the oral feeding performance of preterm infants. Acta Paediatr.2012, 101, e269–e274.	Outcomes were days from start to independent oral feeding, feeding performance etc. No breastfeeding outcomes
20	Lucas, A.; Bishop, N.J.; King, F.J.; Cole, T.J. Randomised trial of nutrition for preterm infants after discharge.Arch. Dis. Child. 1992, 67, 324–327.	Outcomes were growth related, feed tolerance, feed intake etc

21	Manea, A.; Boia, M.; Iacob, D.; Dima, M.; Iacob Radu, E. Benefits of early enteral nutrition in extremely low birth weight infants. Singap. Med. J. 2016, 57, 616–618.	Outcomes were weight gain and feeding intolerance
22	Morlacchi, L.; Mallardi, D.; Giannì, M.L.; Roggero, P.; Amato, O.; Pasqua, P.; Consonni, D.; Mosca, F. Is targeted fortification of human breast milk an optimal nutrition strategy for preterm infants? An interventional study.J. Transl. Med. 2016, 14, 195.	Outcomes were anthropometric indicators
23	Morlacchi, L.; Roggero, P.; Giannì, M.L.; Bracco, B.; Porri, D.; Battiato, E.; Menis, C.; Liotto, N.; Mallardi, D.; Mosca, F. Protein use and weight-gain quality in very-low-birth-weight preterm infants fed human milk or formula. Am. J. Clin. Nutr.2018, 107, 195–200.	Outcomes were on anthropometry and body composition.
24	Morley, R.; Lucas, A. Randomized diet in the neonatal period and growth performance until 7.5-8 y of age in preterm children. Am. J. Clin. Nutr.2000, 71, 822–828.	Outcomes were anthropometric outcomes measured at 9m, 18 mo, and 7.5-8 yrs. No outcome measured at 6 m or before. No breastfeeding outcomes
25	Mosqueda, E.; Sapiegiene, L.; Glynn, L.; Wilson-Costello, D.; Weiss, M. The early use of minimal enteral nutrition in extremely low birth weight newborns. J. Perinatol.2008, 28, 264–269.	Clinical outcomes and feed intolerance were the outcomes
26	Moya, F.; Sisk, P.M.; Walsh, K.R.; Berseth, C.L. A new liquid human milk fortifier and linear growth in preterm infants. Pediatr2012, 130, e928–e935.	Outcomes were on the anthropometry (rate of weight gain, length gain (linear growth), achieved growth (weight, length, and head circumference), ponderal index), enteral/ parenteral intake, feeding tolerance, respiratory status, oliguria, serum chemistries, blood gases, incidence of NEC and sepsis.
27	O'Connor, D.L.; Gibbins, S.; Kiss, A.; Bando, N.; Brennan-Donnan, J.; Ng, E.; Campbell, D.M.; Vaz, S.; Fusch, C.; Asztalos, E.; et al. Effect of Supplemental Donor Human Milk Compared With Preterm Formula on Neurodevelopment of Very Low-Birth-Weight Infants at 18 Months: A Randomized Clinical Trial. J. Am. Med. Assoc. 2016, 316, 1897–1905.	Outcomes were on cognitive composite score on the Bayley Scales of Infant and Toddler Development, Third Edition (Bayley-III) at 18 months' corrected age, Bayley-III language and motor composite scores, a mortality and morbidity index, and

		growth during the feeding intervention.
28	Porcelli, P.; Schanler, R.; Greer, F.; Chan, G.; Gross, S.; Mehta, N.; Spear, M.; Kerner, J.; Euler, A.R. Growth in human milk-Fed very low birth weight infants receiving a new human milk fortifier. Ann. Nutr. Metab. 2000,44, 2–10.	Outcomes were enteral milk intake, anthropometric, biochemical outcomes.
29	Salas, A.A.; Li, P.; Parks, K.; Lal, C.V.; Martin, C.R.; Carlo, W.A. Early progressive feeding in extremely preterm infants: A randomized trial. Am. J. Clin. Nutr. 2018, 107, 365–370.	Outcomes were on growth, full enteral feeding
30	Serrao, F.; Corsello, M.; Romagnoli, C.; D'Andrea, V.; Zecca, E. The Long-Term Efficacy of a Galactagogue Containing Sylimarin-Phosphatidylserine and Galega on Milk Production of Mothers of Preterm Infants. Breastfeed. Med. 2018, 13, 67–69.	Outcome was human milk availability after discharge. No breastfeeding/ infant breastmilk intake outcomes
31	Shah Sanket, D.; Dereddy, N.; Jones Tamekia, L.; Dhanireddy, R.; Talati Ajay, J. Early versus Delayed Human Milk Fortification in Very Low Birth Weight Infants-A Randomized Controlled Trial. J. Pediatr.2016, 174, 126–131.e1.	Outcomes were days to full feeds and number of days to reach full feeding volume.
32	Taheri Peymaneh, A.; Sajjadian, N.; Fargi Marzieh, A.; Shariat, M. Is early breast milk fortification more effective in preterm infants? A clinical trial: Early and late fortification. J. Perinat. Med. 2017, 45, 953–957.	Outcomes were difference in growth indices and complications (feeding intolerance, sepsis)
33	Thomas, N.; Cherian, A.; Santhanam, S.; Jana, A.K. A randomized control trial comparing two enteral feeding volumes in very low birth weight babies. J. Trop. Pediatr.2012, 58, 55–58.	Outcomes were on growth, biochemical and morbidity indicators.
34	Tillman, S.; Brandon, D.H.; Silva, S.G. Evaluation of human milk fortification from the time of the first feeding: Effects on infants of less than 31 weeks gestational age. J. Perinatol.O. J. Calif. Perinat. Assoc. 2012,32, 525–531.	Outcomes measured were growth at 34 weeks and bone mineral status throughout hospitalization
35	Willeitner, A.; Anderson, M.; Lewis, J. Highly Concentrated Preterm Formula as an Alternative to Powdered Human Milk Fortifier: A Randomized Controlled Trial. J. Pediatr. Gastroenterol. Nutr.2017, 65, 574–578.	Outcomes were day-to-day weight gain, feeding intolerance, NEC stage 2 or 3, suspicion of NEC, and positive blood culture. No breastfeeding outcomes

36	Worrell, L.A.; Thorp, J.W.; Tucker, R.; McKinley, L.T.; Chen, J.; Chng, Y.-M.; Vohr, B.R. The effects of the introduction of a high-nutrient transitional formula on growth and development of very-low-birth-weight infants. J. Perinatol.O. J. Calif. Perinat. Assoc. 2002, 22, 112–119.	Outcomes were anthropometric indicators. No breastfeeding outcomes
37	Zecca, E.; Costa, S.; Barone, G.; Giordano, L.; Zecca, C.; Maggio, L. Proactive enteral nutrition in moderately preterm small for gestational age infants: A randomized clinical trial. J. Pediatr.2014, 165, 1135–1139.e1.	Outcomes were LOS, weight loss, time to regain BW, incidence of metabolic abnormalities, and frequency and duration of IV infusion, and anthropometry. No breastfeeding related outcomes

Exclude on target population (n=3)

#	Study detail	Details
1	Clarke, S.E.; Evans, S.; Macdonald, A.; Davies, P.; Booth, I.W. Randomized comparison of a nutrient-dense formula with an energy-supplemented formula for infants with faltering growth. J. Hum. Nutr.Diet. 2007,20, 329–339.	Target population includes congenital cardiac defects, cystic fibrosis and various other organic causes of faltering growth (including intestinal disorders requiring surgery, neurological syndromes and faltering growth of unknown origin.
2	Flaherman, V.J.; Aby, J.; Burgos, A.E.; Lee, K.A.; Cabana, M.D.; Newman, T.B. Effect of early limited formula on duration and exclusivity of breastfeeding in at-risk infants: An RCT. Pediatrics 2013, 131, 1059–1065.	Included exclusively breastfeeding term infants, 24 to 48 hours old, who had lost 5% birth weight. The infants did not have any difficulties in breastmilk intake
3	Slusher, T.; Slusher, I.L.; Biomdo, M.; Bode-Thomas, F.; Curtis, B.A.; Meier, P. Electric breast pump use increases maternal milk volume in African nurseries. J. Trop. Pediatr.2007, 53, 125–130.	Population includes infants who are normal, term birth

Exclude on intervention (n=1)

#	Study detail	Detail
1	Héon, M.; Goulet, C.; Garofalo, C.; Nuyt Anne, M.; Levy, E. An Intervention to Promote Breast Milk Production in Mothers of Preterm Infants. West. J. Nurs. Res. 2016, 38, 529–552.	Intervention included multiple modalities such as breastmilk expression education

		and support intervention to look into volume, duration and frequency of breast milk expression.

Exclude on comparison (n=1)

#	Study detail	Detail
1	Kliethermes, P.A.; Cross, M.L.; Lanese, M.G.; Johnson, K.M.; Simon, S.D. Transitioning preterm infants with nasogastric tube supplementation: Increased likelihood of breastfeeding. J. Obs. Gynecol. Neonatal Nurs.1999, 28, 264–273.	Compares nasogastric tube feeding with bottle feeding

Table S2: Risk of bias assessments

Risk of bias assessment for randomised controlled trials

Study ID	Experimental	Comparator	Outcome	D1	D2	D3	D4	D5	Overall
John	NNS Breast + Standard Care	NNS Finger (Standard Care)	PIBBS total score	?	-	-	+	?	-
John	NNS Breast + Standard Care	NNS Finger (Standard Care)	EBF at 6 weeks	?	-	-	+	?	-
John	NNS Breast + Standard Care	NNS Finger (Standard Care)	EBF at 3 months	?	-	-	+	?	-
John	NNS Breast + Standard Care	NNS Finger (Standard Care)	EBF at 6 months	?	-	-	+	?	-
John	NNS Breast + Standard Care	NNS Finger (Standard Care)	Weight at discharge/ 2m	?	-	-	+	?	-
Fucile	NNS Breast	NNS Pacifier	EBF at discharge/ 1.5 m	?	+	+	+	+	?
Çalıkuşu	Supplemental Feeding Tube Device	Bottle Feeding	LATCH score	?	+	+	?	?	?
Çalıkuşu	Supplemental Feeding Tube Device	Bottle Feeding	Time to transition to full breastfeeding	?	+	+	?	?	?
Çalıkuşu	Supplemental Feeding Tube Device	Bottle Feeding	Daily weight gain	?	+	+	+	?	?
Kumar	Spoon Feeding	Nasogastric Tube Feeding	Time to transition to breastfeeding	+	?	+	-	?	-
Kumar	Spoon Feeding	Nasogastric Tube Feeding	Weight at 7th day	+	?	+	+	?	?
Kumar	Spoon Feeding	Nasogastric Feeding Tube	Weight at 14th day	+	?	-	+	?	-
Kumar	Spoon Feeding	Nasogastric Tube Feeding	Weight gain from birth to transition to full breastfeeding	+	?	+	+	?	?
Yilmaz	Cup Feeding	Bottle Feeding	EBF at discharge	+	-	-	-	?	-
Yilmaz	Cup Feeding	Bottle Feeding	EBF at 3 months	+	-	-	-	?	-
Yilmaz	Cup Feeding	Bottle Feeding	EBF at 6 months	+	-	-	-	?	-
Yilmaz	Cup Feeding	Bottle Feeding	Weight gain in first 7 days of study	+	-	-	-	?	-

D1: bias arising from randomisation process; D2: bias due to deviations from intended interventions; D3: bias due to missing outcome data; D4: bias in measurement of the outcome; D5: bias in selection of the reported result
EBF: exclusive breastfeeding; LATCH: Latch, Audible swallow, Type of nipple, Comfort, Hold; m: months; PIBBS: Preterm Infant Breastfeeding Behaviour Scale; m: months; NNS: non-nutritive sucking; red: high risk; yellow: some concerns; green: low risk

Risk of bias assessment for non-randomised controlled trials

Author (year)	Outcome	Pre-intervention		At intervention	Post-intervention				Overall risk of bias
		Bias due to confounding	Bias in selection of participants into the study	Bias in classification of interventions	Bias due to deviations from intended interventions	Bias due to missing data	Bias in measurement of outcomes	Bias in selection of the reported result	
Abouelfettoh (2008)	EBF at 1 week post discharge	Not assessed	Low	Serious	Low	Low	Serious	Low	Serious
Say (2019)	Time to transition to breastfeeding	Not assessed	Moderate	Serious	No information	Low	No information	No information	Serious
Say (2019)	Breastfeeding at 6 months	Not assessed	Moderate	Serious	No information	Low	No information	No information	Serious
Say (2019)	Daily weight gain	Not assessed	Moderate	Serious	No information	Low	No information	No information	Serious
Say (2019)	Weight at discharge	Not assessed	Moderate	Serious	No information	Low	No information	No information	Serious

EBF: exclusive breastfeeding

Table S3: Detailed result table

Author (Year)	Study Design	Sample size	Intervention	Comparison	Breastfeeding Outcome	Anthropometry
Say (2019)	N-RCT	103 (IG=47, CG=56)	**Syringe feeding** with BM or formula	**Bottle feeding** with NUK nipple	**Time to transition to BF (post-natal age in days) (Mean, SD)**<br>IG: 42.54 ± 21.21,<br>CG: 50.45 ± 15.95, p=0.04<br>**Breastfeeding at 6 months post discharge, (n/N)**<br>IG: 46 (46.5)/47,<br>CG: 53(53.5)/56, p=0.38	**Weight at discharge(g) (Mean, SD)**<br>IG: 2027.89±357.3,<br>CG: 2050.23±434.8, p=0.77<br>**Daily weight gain(g/d) (Mean, SD)**<br>IG: 22.11±12.61,<br>CG: 20.19±7.20, p=0.33
Çalıkuşu (2021)	RCT	46 (IG=23, CG=23)	**Supplemental Feeding Tube Device**	**Bottle feeding** by mothers	**LATCH Score at discharge (Mean, SD)**<br>IG: 9.87±0.34, CG:9.74±0.54, p=0.413<br>**Transition to full BF (days) (Mean, SD)**<br>IG: 4.70±2.44, CG: 6.00±4.10, p>0.05	**Daily weight gain (g) (Mean, SD)**<br>IG: 24.09±15.21,<br>CG: 27.17 ± 17.63 g, p> 0.05
Kumar (2010)	RCT	79 (IG=40, CG=39)	**Spoon feeding** by mother/nurse in hospital	**Nasogastric tube** by nurse only	**Time to transition to breastfeeding (days) (Mean, SD)**<br>IG: 14.39±4.10 (n=40),<br>CG: 12.31±3.32 (n=39), p=0.02	**Weight at 7th day of life (g) (Mean, SD)**<br>IG: 1393.72 ±111.72 (n=39),<br>CG: 1411.80±113.70 (n=39)<br>**Weight at 14th day of life (g) (Mean, SD)**<br>IG: 1492.50±113.12 (n=37),<br>CG: 1489.29±120.42 (n=37)<br>**Weight gain from birth till transition to full BF (g/kg/d) (Mean, SD)**<br>IG: 4.47±3.14,<br>CG: 4.72±4.68, p=0.88
Yilmaz (2014)	RCT	607 (IG=308, CG=299)	**Cup feeding** using medicine cup, 15 ml of breast	**Bottle feeding** with teat/nipple, breastmilk/ formula	**EBF at discharge, (n/N)**<br>(IG: 184/254,CG:123/268)<br>**EBF at 3 month, (n/N)**<br>(IG:196/254,CG: 126/268)	**Weight gain in the first 7 days of study (g/d) (Mean, SD),**<br>IG: 16.7 ± 1.5, CG=16.8 ± 1.5, p= 0.64

Author (Year)	Study Design	Sample size	Intervention	Comparison	Breastfeeding Outcome	Anthropometry
			milk/ formula given		**EBF at 6 month, (n/N)**<br>(IG: 146/254, CG: 113/268)	
Abouelfettoh (2008)	N-RCT	60 (IG= 30, CG= 30)	**Cup feeding** was given by PI or nurse	**Bottle feeding** was given by nurse or PI (given first, before intervention)	**EBF at 1 week post discharge (%)(n/N)**<br>IG: 14/30, CG: 10/30	
John (2019)	RCT	14 (IG=7, CG=7)	**Suckling on emptied breast** for 5-10 mins, 3 times a day + Standard Care	**NNS on finger** (standard care)	**PIBBS Total score (Out of 20) at discharge**<br>IG:14.2±1.7 ; CG: 10.2±4, p=0.19<br>**EBF at 6 weeks (n/N)**<br>IG=3/4 vs CG= 5/5<br>**EBF at 3 months,(n/N)**<br>IG=2/4 vs CG= 5/5<br>**EBF at 6 mo,(n/N)**<br>IG=1/4 vs CG=3/5	**Weight at discharge (g) (Mean, SD)**<br>IG: 1910 ± 142,<br>CG: 1935 ± 127.9, p= 0.78
Fucile (2021)	RCT	33 (IG= 17, CG=16)	**NNS on emptied breast** once a day for 15 min	**NNS on a pacifier** once a day for 15 minutes	**EBF at discharge(n/N)**<br>IG=10/16 vs CG= 4/17, p=0.03	

Notes: RCT= randomised control trial, NRCT= non-randomized control trial, NNS= Non nutritive sucking,EBF= exclusive breastfeeding, IG= intervention group, CG= comparision group, SD= standard deviation, LATCH= Latch, Audible swallowing, Type of nipple, Comfort, Hold) total score; PIBBS (Preterm Infants Breastfeeding Behaviour Scale , g= grams, kg= kilograms
